# Quantitative *N-*Glycan Profiling of Therapeutic Monoclonal Antibodies Performed by Middle-Up Level HILIC-HRMS Analysis

**DOI:** 10.3390/pharmaceutics13111744

**Published:** 2021-10-20

**Authors:** Bastiaan L. Duivelshof, Steffy Denorme, Koen Sandra, Xiaoxiao Liu, Alain Beck, Matthew A. Lauber, Davy Guillarme, Valentina D’Atri

**Affiliations:** 1 Institute of Pharmaceutical Sciences of Western Switzerland (ISPSO), University of Geneva, CMU—Rue Michel-Servet 1, 1211 Geneva, Switzerland; Bastiaan.Duivelshof@unige.ch (B.L.D.); Davy.Guillarme@unige.ch (D.G.); 2 School of Pharmaceutical Sciences, University of Geneva, CMU—Rue Michel-Servet 1, 1211 Geneva, Switzerland; 3 Research Institute for Chromatography (RIC), President Kennedypark 26, 8500 Kortrijk, Belgium; steffy.denorme@RIC-group.com (S.D.); koen.sandra@RIC-group.com (K.S.); 4 Waters Corporation, 34 Maple Street, Milford, MA 01757-3696, USA; Xiaoxiao_Liu@waters.com (X.L.); Matthew_lauber@waters.com (M.A.L.); 5 IRPF—Centre d’Immunologie Pierre-Fabre (CIPF), 5 Avenue Napoléon III, 60497 Saint-Julien-en-Genevois, France; alain.beck@pierre-fabre.com

**Keywords:** glycan profiling, quantitative analysis, HILIC-MS, protein subunits, monoclonal antibodies

## Abstract

The identification and accurate quantitation of the various glycoforms contained in therapeutic monoclonal antibodies (mAbs) is one of the main analytical needs in the biopharmaceutical industry, and glycosylation represents a crucial critical quality attribute (CQA) that needs to be addressed. Currently, the reference method for performing such identification/quantitation consists of the release of the *N*-glycan moieties from the mAb, their labelling with a specific dye (e.g., 2-AB or RFMS) and their analysis by HILIC-FLD or HILIC-MS. In this contribution, the potential of a new cost- and time-effective analytical approach performed at the protein subunit level (middle-up) was investigated for quantitative purposes and compared with the reference methods. The robustness of the approach was first demonstrated by performing the relative quantification of the glycoforms related to a well characterized mAb, namely adalimumab. Then, the workflow was applied to various glyco-engineered mAb products (i.e., obinutuzumab, benralizumab and atezolizumab). Finally, the glycosylation pattern of infliximab (Remicade^®^) was assessed and compared to two of its commercially available biosimilars (Remsima^®^ and Inflectra^®^). The middle-up analysis proved to provide accurate quantitation results and has the added potential to be used as multi-attribute monitoring method.

## 1. Introduction

Recombinant monoclonal antibodies (mAbs) serve a fundamental role in the field of human therapeutics by providing highly efficacious therapies in crucial disease areas, such as oncology, auto-immune and skin diseases [1]. Inherent to their manufacturing in cellular expression systems is the occurrence of numerous enzymatic and chemical post-translational modifications (PTMs) [2]. Glycosylation is considered to be one of the most critical PTMs due to its major role in the stability, immunogenicity and the clinical efficacy of the mAbs [3]. Most IgG-type mAbs contain a conserved *N*-glycosylation site, located in the crystallizable fragment (Fc) carrying oligosaccharide structures of a high-mannose, hybrid or complex type structure, depending on the cellular expression system. It has been shown that these distinct glycan motifs are highly heterogeneous and can significantly influence important Fc-mediated effector functions, such as complement-dependent cytotoxicity (CDC), antibody-dependent cellular cytotoxicity (ADCC) and selective antibody clearance [4,5]. Furthermore, several distinct glycoforms, e.g., *N*-glycolylneuraminic acid or α1,3-bound galactose containing glycans, are associated with adverse immunogenic reactions [6,7]. Taken together, this renders the glycan profile an important critical quality attribute (CQA) that requires comprehensive characterization to ensure safe and efficacious treatments for patients.

With the recent shift towards the development of glyco-engineered mAbs and the rapidly emerging biosimilar market, there is an emerging need for strong analytical techniques that enable accurate glycan characterization from research and development to industrial-scale bioprocessing [8,9]. However, in the absence of a direct genomic blueprint, the characterization of the glycan profile remains challenging. Current methods commonly rely on the enzymatic release of the *N*-glycans from the protein using, e.g., peptide-*N*-glycosidase F (PNGase F) to enable the analysis of the glycans separately from the protein. To detect the released oligosaccharide structures, the glycans are derivatized to add a chromophore to the carbohydrate structures and to enable spectroscopic detection techniques. The use of the 2-aminobenzamide (2-AB) label is widely considered as a reference derivation procedure prior to hydrophilic interaction liquid chromatography (HILIC) [10,11]. The labelled glycans can then be easily separated and detected using fluorescence detection (FLD) and characterized by converting the retention times for each glycan to glucose units (GU), which is a measurement that reduces instrument-to-instrument and lab-to-lab variability. The obtained GU values can be compared to publicly available databases as a means to make preliminary peak identifications. However, labelled glycan approaches are often long and laborious procedures with overnight enzymatic incubations and multi-hour labelling reactions [12]. In addition, labelling agents such as 2-AB are often responsible for poor ionization efficiencies in electrospray ionization (ESI)-mass spectrometry (MS). 2-AB is also used with acidic reductive amination reactions that can cause desialylation. Therefore, the characterization of only pre-determined glycans is possible and the accurate identification is dependent on the baseline separation of the glycans. Fortunately, new labelling agents, such as RapiFluor-MS (RFMS) or InstantPC™, have been developed with improved ionization efficiencies and significantly quicker labelling procedures [13,14,15]. This allows an increased sample preparation throughput and sensitive MS measurements that can provide accurate glycan identification and confirmation. Nevertheless, the released glycan approaches do not provide site-specific information and are unable to detect other important PTMs present on therapeutic mAbs.

Recently, the use of HILIC-MS at protein subunit level has emerged as a powerful technique for the qualitative glycan analysis of mAbs, biosimilars, fusion proteins and ADC products [16,17,18,19,20,21,22,23]. Protein subunits can be simply obtained after enzymatic digestion using specific proteases (e.g., *IdeS*, Papain) and the chemical reduction in the disulfide bonds in a short (±1 h) and easily automatable sample preparation procedure [24,25]. Then, with the use of wide-pore (300 Å) HILIC stationary phases, the highly informative mAb subunits, of approximately 25 kDa, can be well separated and provide important site-specific information on the glycosylation profile, as well as other important PTMs [21,23]. Contrary to RPLC based methods, HILIC can chromatographically resolve scFc glycoforms before introduction to ESI-MS. This allows more accurate peak deconvolution and prevents negative effects on the ionization process when multiple species arrive simultaneously in the ionization source (matrix effects).

Middle-up LC-MS analysis holds significant potential to be used as a multi-attribute monitoring method (MAM) that could complement laborious released glycan approaches and complex MAM peptide-mapping workflows [26,27]. Despite the potential advantages, middle-up HILIC-MS analysis for *N*-glycan quantification has not yet been widely explored.

In this study, we set out to evaluate the performance of middle-up HILIC-MS analysis for the identification and quantification of *N*-glycans of therapeutic mAbs. In this context, different therapeutic mAb products with distinct glycosylation profiles (e.g., low fucose, bisecting and biosimilar products) are selected and analyzed using HILIC-MS performed at the subunit level and by using the released *N*-glycan approach as a reference method. First, adalimumab, a well characterized mAb, is analyzed to compare the relative abundance levels obtained from the subunit mass spectra with the glycan abundance levels obtained from 2-AB and RFMS released glycan profiling. Finally, to demonstrate the potential of the quantitative middle-up HILIC-MS approach for more complex mAb samples, the middle-up approach is applied to multiple glyco-engineered mAb products as well as two biosimilar products versus its innovator counterpart.

## 2. Materials and Methods

### 2.1. Chemical and Reagents

Acetonitrile (ACN), methanol (MeOH) and water were LC-MS grade (Optima^®^) and obtained from Fisher Chemical (Reinach, Switzerland). Formic acid (ULC-MS, FA, 99%) and trifluoroacetic acid (ULC-MS, TFA, >99.0%) were obtained from Biosolve BV (Valkenswaard, The Netherlands). DL-dithiothreitol (DTT, >99%) was obtained from Axon Lab AG (Baden, Switzerland). Ammonium bicarbonate (>99.5%), iodoacetamide (IAA, BioUltra), ammonium citrate tribasic (>97%), acetic acid glacial, DMSO, 2-aminobenzamide (2-AB), sodium cyanoborohydride and ammonium formate (MS-grade) were obtained from Sigma-Aldrich (Buchs, Switzerland). TRIS-HCl buffer 1M pH 8.0 (UltraPure) was obtained from Thermo Invitrogen (Waltham, MA, USA). PNGase F (500,000 U/mL, P0704S) was obtained from New England BioLabs (Ipswich, MA, USA). *IdeS* enzyme (FabRICATOR^®^) was purchased from Genovis AB (Lund, Sweden). Rapigest SF surfactant, GlycoWorks HILIC µElution plate and a Glycoworks RapiFluor-MS 24-sample *N*-glycan kit were obtained from Waters (Milford, MA, USA). The kit provided all the required chemicals and reagents to complete the sample preparation for RFMS-labelled *N*-glycans. Adalimumab, obinutuzumab, benralizumab, atezolizumab and infliximab (Remicade^®^, Inflectra^®^, Remsima^®^) were obtained as European Union pharmaceutical-grade drug products from their respective manufacturers.

### 2.2. Sample Preparation

#### 2.2.1. 2-AB Glycan Labelling

For 2-AB labelling, the sample preparation protocol included several enzymatic steps, namely, mAb denaturation, reduction and deglycosylation followed by glycan purification, labelling and a second purification step. First, the protein material was dissolved in a 1% solution of Rapigest surfactant and incubated with 5 mM DTT for 30 min at 60 °C. Then, to prevent the reformation of disulfide bonds, cysteine alkylation with 10 mM IAA was performed for 30 min in the dark at RT. mAb *N*-deglycosylation was then performed by incubating 57 µg of protein material with 2.98 µL of PNGase F (1.91 U/mL) for 18 h at 37 °C. The released *N*-glycans were recovered using a HILIC-SPE µElution plate (Waters) after washing (90% ACN), elution (1 mM Ammonium Tris Citrate in 10% ACN) and evaporation to dryness. Labelling of the released *N*-glycans was performed by incubation with the 2-AB dye (dissolved in 30% acetic acid in DMSO) at 65 °C for 3 h in the dark. Then, excess dye was removed in a second identical HILIC-SPE procedure followed by evaporation to dryness. The dried samples were stored in the dark at −20 °C prior to analysis.

#### 2.2.2. RFMS Glycan Labelling

The enzymatic release and RFMS labelling of *N*-glycans was performed according to the Waters Glycoworks RapiFluor-MS *N*-Glycan Kit Care and Use Manual [28]. The protocol was carried out by introducing a glycoprotein quantity of 15 µg for each antibody product. The obtained RFMS derivatized *N*-glycans were directly analysed after labelling. For the RFMS label, approximately 5 pmol of labelled glycan material was loaded onto the column.

#### 2.2.3. Protein Subunit Generation

mAb protein subunits were generated by adding 120 units of *IdeS* enzyme to 120 µg of mAb in a solution of 10 mM Tris buffer (pH 7.3). The final concentration of 2 mg/mL was incubated for 30 min at 45 °C. Consequently, 100 mM of DTT was added to the digested material and incubated for 30 min at 45 °C. The sample material was directly analysed after the subunit generation.

### 2.3. Instrumentation and Columns

All experiments were performed on an ultra-high-performance liquid chromatography (UHPLC) system (Acquity UPLC I-Class Plus, Waters Milford, MA, USA) coupled to a time-of-flight (TOF) mass spectrometer (BioAccord Acquity RDa, Waters Milford, MA, USA). The UHPLC system was equipped with a binary solvent delivery pump, flow-through needle (FTN) sample manager and a fluorescence detector (FLD) and operated using UNIFI Software (v1.9.9.3. Waters, Milford, MA, USA). The FLD settings were specific for each application. For subunit analysis, the settings were: λ_ex_ = 280 nm and λ_em_ = 360 nm, 10 Hz; for RFMS labelled glycans, the settings were: λ_ex_ = 265 nm and λ_em_ = 425 nm, 2 Hz; for 2-AB labelled glycans, the settings were: λ_ex_ = 330 nm and λ_em_ = 420 nm, 10 Hz.

For HILIC-MS experiments of subunits, the MS device was operated in ESI+ mode with a capillary voltage of 1.5 kV, a desolvation temperature of 550 °C and a cone voltage of 120 V. Full scan acquisition was performed with Intelligent Data Capture (IDC) on and a mass range of 400–7000 *m*/*z* with a scan rate of 2 Hz.

For HILIC-MS analysis of the RFMS labelled glycans, the MS was operated in ESI+ mode with a capillary voltage of 1.5 kV, desolvation temperature of 300 °C and a cone voltage of 45 V for full scan and 70–90 V for fragmentation. The acquisition was performed with IDC on in the range of 50–2000 *m*/*z* with a scan rate of 2 Hz. For 2-AB labelled glycans, identical MS settings were applied, except no fragmentation was performed. The system was calibrated by using sodium iodide (2 µg/µL in 50% isopropanol) and a mixture of leucine enkephalin (150 pg/µL), caffeine (500 pg/µL) and pentanesulfonic acid (100 pg/µL) in 50/50 ACN/H_2_O with 0.1% FA was used as a lock mass reference.

Waters Acquity UPLC GlycoProtein Amide (1.7 µm, 150 mm × 2.1 mm, 300 Å) column and Waters Acquity UPLC BEH Amide Glycan (1.7 µm, 150 mm × 2.1 mm, 130 Å) column were used for the subunit and the released *N*-glycans analysis, respectively.

### 2.4. Chromatographic Conditions

The separation of protein subunits in HILIC was performed by using 0.1% TFA water and 0.1% TFA in ACN as mobile phases A and B, respectively. A gradient of 85% to 73% B in 0.2 min, followed by 73% to 65% B in 12 min was applied. Following the analytical gradient, an isocratic step at 15% B for 1 min was used to wash the column, followed by 9 min of column re-equilibration at 85%B. The flow rate was 0.4 mL/min, column temperature 80 °C and injection volume 0.5 µL (2 mg/mL sample material).

For the separation of the 2-AB labelled *N*-glycans, 100 mM ammonium formate (pH 4.5) and 100% ACN were used as mobile phases A and B, respectively. A gradient of 78%B to 55.9%B in 38.5 min was applied at a flow rate of 0.5 mL/min, followed by a column cleaning procedure with 20% B for 5 min and at a reduced flow rate of 0.25 mL/min. Then, the flow rate was increased to 0.5 mL/min for 5.5 min of column re-equilibration at 78%B. The column temperature was 60 °C, and 2.5 µL (38 pmol sample material) was injected. Directly before analysis, the dried sample material was reconstituted in 50:50 ACN/H_2_O solution.

For the RFMS labelled *N*-glycans, 50 mM ammonium formate (pH 4.4) and 100% ACN were used as mobile phases A and B, respectively. A gradient from 75%B to 54%B was run for 35 min at a flow rate of 0.4 mL/min, followed by an isocratic step at 20%B for 3 min at a reduced flow rate of 0.2 mL/min to wash the column. Then, the flow rate was increased back to 0.4 mL/min and the column was re-equilibrated at 75%B. The column temperature was 60 °C and 10 µL of sample diluent (32/68% DMF/ACN) was injected, corresponding to 5 pmol of labelled glycan material.

## 3. Results and Discussion

### 3.1. Comparison of Quantitative N-Glycoprofiling Methods

#### 3.1.1. Released *N*-Glycan Analysis

To obtain reference values for the glycan profile of adalimumab, 2-AB and RFMS, labelled *N-*glycans were generated and analyzed using HILIC-FLD-HRMS. The HILIC analysis was performed in triplicate and the observed peaks corresponding to *N-*glycan species in the FLD chromatogram were identified based on their elution position relative to the dextran calibration ladder (glucose units, GU). Separate dextran calibration ladders were analyzed for the 2-AB and RFMS label and applied independently to calculate the label specific GU values that were used for peak identification, using the publically available GU libraries. Further confirmation of the peak assignments was obtained through the interpretation of the collected MS data. However, due to the limited ionization efficiency of the 2-AB label, only the four most abundant glycans (G0F-N, G0F, G1F and M5) could be confirmed using the MS spectra [13].

**Figure 1:**
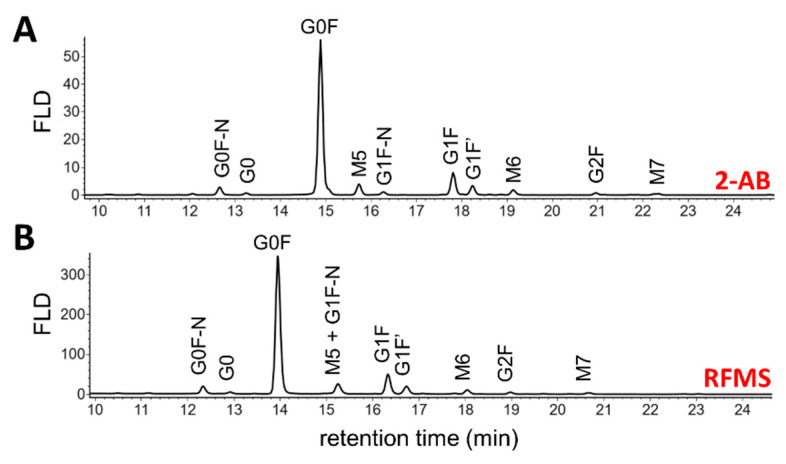
Fluorescence chromatograms of HILIC separated *N-*glycans of adalimumab after 2-AB (**A**) and RFMS (**B**) labelling. Different gradient profiles and mobile phase compositions were used for the HILIC analysis of the 2-AB and RFMS labelled glycans (see Section 2.4). Glycan identification based on calculated GU values and/or MS detection.

Figure 1 shows the released *N-*glycan profiles of adalimumab using both the 2-AB and RFMS labels. It was observed that highly similar profiles were obtained for both methods, with minor differences in selectivity resulting from the implemented mobile phase conditions. RFMS glycan profiling was performed with a 50 mM buffer concentration, versus 100 mM, out of a preference to run analyses with a more MS-friendly condition. Nevertheless, a clear difference in selectivity was only observed for the M5 and G1F-N glycoforms, which were separated when using the 2-AB label and co-eluted when derivatized with the RFMS label. Previous work has shown that an increase in ammonium formate mobile phase concentration, gradient steepness and column temperature can affect glycan selectivity in this and other regions of the elution profile [29,30]. For both labels, minimal deviations in retention times were observed, having RSD values of 0.6% and 1.5% for 2-AB and RFMS, respectively (calculated based on G0F, *n* = 3).

Concerning the glycan profile, predominantly complex-type biantennary species were identified, as generally expected for antibodies produced in mammalian cell culture systems such as CHO cells [31]. Based on the FLD chromatograms, 10 major glycan species were selected (Figure 1) and quantified by calculation of their relative abundance (Table 1). A good correlation between the *N-*glycan abundance levels obtained after 2-AB and RFMS labelling was found, with, e.g., a relative quantification difference of 1.6% for G0F between the two methods. Interestingly, the comparison between the two methods highlighted an overestimation in the relative abundance of the M5 glycoform in the RFMS glycan profile, due to the co-elution with the G1F-N glycoform (Figure 1B). A suggested strategy to correct for the overestimation of the co-eluting M5 and G1F-N RFMS glycans is by using their MS intensities to divide the obtained relative abundance level of the co-eluted peak into two separate percentages for the M5 and G1F-N glycoforms. This allowed for correction of the relative FLD abundance levels of the co-eluted species and, thus, obtained similar values as with the 2-AB labelling method, with 4.7% and 1.3% for M5 and G1F-N, respectively (Figure S1). However, this strategy comes at the cost of introducing a secondary data processing procedure that has to be performed separately from the main workflow. This co-elution was not detected in the 2-AB *N-*glycan profile, which therefore provides a more accurate relative quantification.

Moreover, the 2-AB labelling method, used as a reference technique, showed good precision in the relative abundance levels obtained by FLD and a good correlation with the literature [32,33]. However, due to the limited ionization efficiency of the 2-AB label, it was not possible to characterize and quantify the glycan profile solely based on the MS information (Table 1). It was observed that integration of the MS based detection profiles of the RFMS labelled *N-*glycans showed minor differences with the values obtained with FLD, even though the MS based values themselves showed slightly increased standard deviations. Nevertheless, no clear effects on the relative quantification were observed when using only MS.

#### 3.1.2. Middle-Up HILIC-HRMS Glycan Profiling

**Figure 2:**
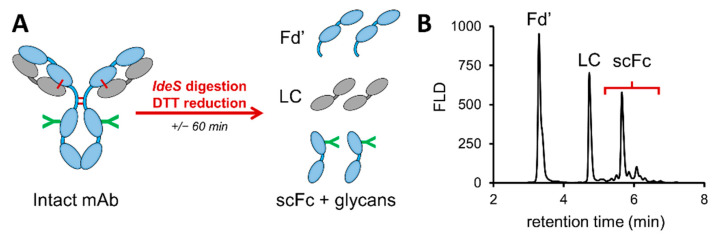
Middle-up HILIC-MS analysis of *IdeS* digested and DTT reduced adalimumab. (**A**) Sample preparation procedure for the digestion and reduction in intact mAb to protein subunits. (**B**) FLD chromatogram displays the separation of the Fd’, LC and scFc subunits. See Table S1 for detailed retention times and mass assignment.

Subunits of adalimumab were generated thanks to a rapid enzymatic digestion with *IdeS* and chemical reduction in the disulfide bonds using DTT. The mAb fragments (Figure 2A) of approximately 25 kDa were then analyzed in HILIC-MS to characterize the main PTMs and quantify the different glycans.

**Figure 3:**
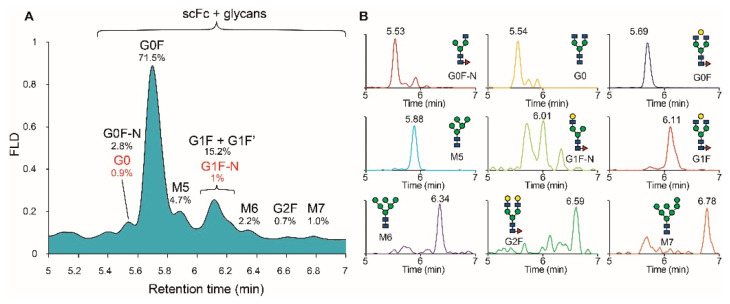
Zoom of the glycosylated scFc subunit of adalimumab in HILIC-MS analysis (**A**,**B**) the extracted ion chromatograms (XIC) of the scFc subunit carrying the identified glycans. Relative glycan abundance levels provided for the scFc subunit carrying the glycoforms and determined based on the integrated XIC profiles (*n* = 3). Glycan nomenclature: *N-*acetyl glucosamine (blue), fucose (red), mannose (green), galactose (yellow).

Figure 2B illustrates the obtained HILIC separation of the Fd’, LC and scFc subunits, with the latter containing the *N-*glycosylation sites. The analyses were performed in triplicate and demonstrated minimal deviations in retention times, with a RSD value of 0.6% for the LC subunit (*n* = 3). MS peak identification allowed the accurate mass determination of the Fd’ and LC subunits (Table S1). Moreover, deconvolution of the mass spectra showed that the peak eluting at 5.12 min could be attributed to the LC fragment with a mass shift of + 162 Da, indicating a glycated variant of the LC. The scFc subunit was chromatographically resolved in multiple peaks corresponding to different scFc subunits carrying different *N-*glycan species. Thanks to the possibility of convoluting each peak separately, a more accurate identification of each glycoform was performed by using MS (Table S1). However, based on the peaks observed in the chromatogram, only eight different glycan species were identified (Figure 3A).

This is due to the co-elution of the G0 and G1F-N glycoforms with the G0F-N and G1F/G1F’ glycoforms, respectively. As a result, performing a direct relative quantification based on the FLD chromatogram will provide inaccurate abundance levels for the aforementioned glycan species (Table 1). To avoid this problem, we performed a relative quantification based on the summation of the extracted ion chromatograms (XIC) of three different charge states of the ions corresponding to the scFc subunits carrying different glycoforms.

To obtain the XICs for each scFc glycoform, the theoretical mass of each glycan was added to the mass of the scFc subunit and divided by the amount of charges present on the ion. Then, the charge states of each glycoprotein were extracted from the MS spectra with a 0.5 Da tolerance. The calculation of relative abundance levels was then performed by comparison of the summed response values. For the scFc subunits of adalimumab, it was observed that the most abundant ions in the mass spectra were carrying 12–14 charges. Therefore, these charge states were extracted from the total ion chromatogram (TIC) for each of the expected scFc glycoforms and used to create the different summed XIC profiles (Table 2 and Figure S2).

Figure 3B illustrates the XICs of the different scFc glycoforms found in adalimumab. From the chromatograms, the individual retention times for each scFc glycoform can be derived accurately with, e.g., a RSD of 0.6% for M5 (*n* = 3). Moreover, the individual evaluation of the different scFc glycoforms allowed co-eluting species G1F and G1F-N to be distinguished (Table 2). For the G0 and G0F-N glycoforms, it was observed that no distinction could be made based on the retention time. Fortunately, by using their differences in mass, two independent XIC profiles could be extracted and used to calculate the relative abundance levels of each glycan species separately (Table 2). For both the co-eluting glycan species, the XIC based relative quantification allowed FLD abundance levels to be corrected from 8.3% to 4.7% and 4.6% to 2.8% for M5 and G0F-N, respectively. In addition, the relative quantification of the G0 and G1F-N glycoforms was finally feasible by using the MS-based approach (Table 1). This clearly demonstrated the potential of using this MS-based approach for relative glycoform quantification, as opposed to quantification by FLD alone. Moreover, the relative abundance levels that were obtained from the XIC showed excellent precision, with RSD values under 0.5% for all detected scFc glycoforms.

To evaluate the accuracy of the relative glycan quantitation at the subunit level, the glycan abundance levels were compared to the reference 2-AB method. It was observed that minimal differences exist in relative abundance levels between the two methods for all present glycoforms (Table 1). Moreover, a good fit was observed between the values for several important *N-*glycan characteristics that can be considered as CQAs. The sum of high mannose containing species (M5, M6, M7) was 7.4% and 7.9% for the 2-AB and middle-up approach, respectively. For the galactosylated species (G1F, G1F’, G1F-N, G2F), the summed amounts were 16.9% for both approaches. At last, both techniques quantified the G0 glycoform at 0.9%, which corresponds to the afucosylated species in adalimumab. Since little differences were observed between the 2-AB and RFMS based methods, a similar correlation exists between the results obtained on subunit level and the results obtained with the RFMS method after correction of the relative abundance levels for the co-eluted glycoforms.

Overall, the results demonstrated the strong quantitation potential of the XIC approach at the middle-up level, together with a good correlation with the reference 2-AB method. In addition, the subunit analysis enabled the detection of other PTMs, such as lysine clipping and glycation (Table S1), and therefore offers even more potential because of its utility as a multi-attribute monitoring approach.

### 3.2. Application of Quantitative Middle-Up Analysis to Diverse mAb Material

To further evaluate the quantitative performance of the middle-up approach for glycan analysis, a variety of diverse mAb materials were analyzed. For this purpose, mAbs with different *N-*glycan characteristics were selected, such as the glyco-engineered products atezolizumab (aglycosylated), benralizumab (low fucose) and obinutuzumab (bisecting and low fucose). In addition, the innovator product of infliximab (Remicade^®^) was compared with two biosimilar products (Remsima^®^ and Inflectra^®^) that have known differences in the *N-*glycosylation profile [33,34,35,36]. It is worth noting that this set of samples included mAbs produced in different cell lines, namely mammalian CHO (adalimumab, atezolizumab, benralizumab, obinutuzumab) and murine SP2/0 (infliximab).

**Figure 4:**
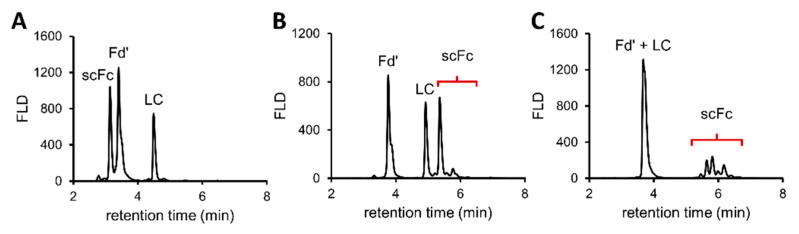
Middle-up HILIC-MS analysis of *IdeS* digested and DTT reduced atezolizumab (**A**), benralizumab (**B**) and obinutuzumab (**C**). FLD chromatograms display the separation of the Fd’, LC and scFc subunits. See Tables S2 and S3 for detailed retention times and mass assignments.

Figure 4 details the FLD profiles of the different glyco-engineered products at the subunit level. With the aglycosylated atezolizumab (Figure 4A), it was observed that the absence of glycans on the scFc subunit greatly reduced the retentivity of this fragment on the HILIC column, such that elution of the scFc subunit occurred before the Fd’ and LC subunits. A similar but smaller effect was observed for the scFc of benralizumab (Figure 4B), where a decrease in retention can be attributed to the absence of fucosylated glycan species due to the special production of the mAb in 1,6-fucosyl transferase knockout CHO cells [37]. This was confirmed by the relative quantification of the glycan composition that demonstrated the presence of exclusively afucosylated complex biantennary and high mannose glycan species (Table S2).

For obinutuzumab (Figure 4C), it was observed that the Fd’ and LC subunits co-eluted, but multiple chromatographically resolved scFc glycoforms could be identified. The identification and relative quantification of the scFc glycoforms showed that ~67% of the glycans contained bisecting GlcNac, and 50% was afucosylated (Table S3). This was in accordance with the fact that this mAb was expressed from cells specifically designed to create bisected *N-*glycans that cannot be core-fucosylated during the production process, and therefore have increased ADCC activity [37,38]. In conclusion, the middle-up approach was exceptionally well suited for the identification and quantification of important *N-*glycan characteristics of glyco-engineered products.

**Figure 5:**
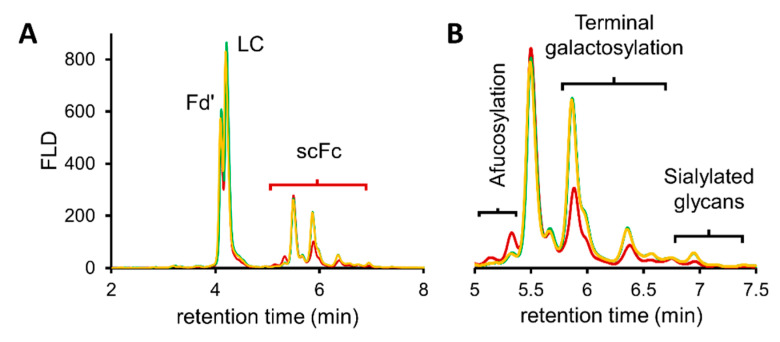
HILIC-MS analysis of infliximab products Remicade^®^, Remsima^®^ and Inflectra^®^. (**A**) Fluorescence chromatograms of the glycosylated scFc subunit of Remicade^®^ (red), Remsima^®^ (green) and Inflectra^®^ (yellow). (**B**) Zoom on the scFc subunit region with sections assigned for scFc subunits carrying afucosylated glycans, terminal galactosylated glycans and sialylated glycans.

Another important field of application was investigated, consisting of comparability studies between originator and biosimilar mAb products, as shown in Figure 5. Figure 5B illustrates the FLD profiles of the scFc subunit of Remicade^®^ (originator), Remsima^®^ and Inflectra^®^ (biosimilars). At even the chromatographic level, it can be observed that differences exist between the innovator and its biosimilar counterparts, most especially in terms of afucosylation, terminal galactosylation and sialylation.

**Figure 6:**
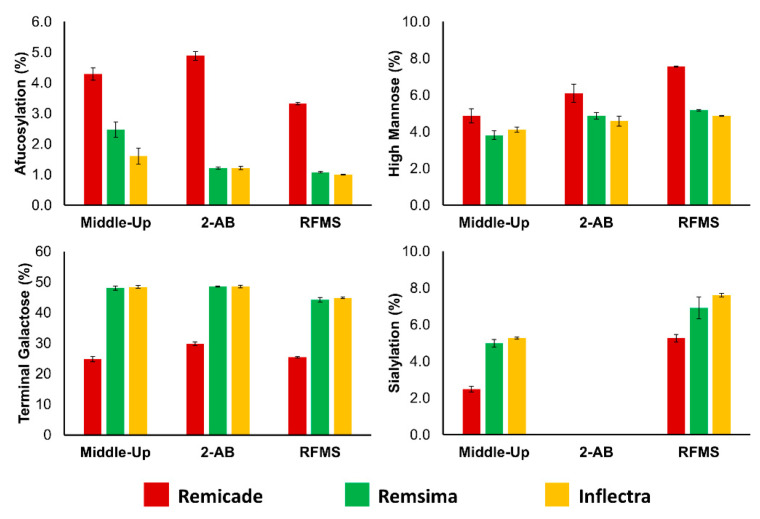
Comparison of *N-*glycan characteristics of infliximab products. Relative quantification performed with FLD for 2-AB and RFMS labeled *N-*glycans and MS detection for middle-up analysis of Remicade^®^, Remsima^®^ and Inflectra^®^. Afucosylation corresponds to the sum of the G0, G0-N, G1 afucosylated glycans. Sialylation corresponds to the sum of all sialylated glycans. Galactosylation corresponds to the sum of terminal galactosylated glycans. High mannose represents the M5 glycan species. Relative abundance levels provided in percentages with standard deviations (*n* = 3).

Similar differences between the products were found after released glycan analysis with 2-AB (Figure S3) and RFMS (Figure S4) released glycan profiling. A comparison of these glycan data is displayed in Figure 6. It was observed that afucosylation is relatively high in Remicade^®^ and that both biosimilar products contained more glycan species with terminal galactosylation. Moreover, a higher level of sialylation was found for Remsima^®^ and Inflectra^®^, while slightly increased levels of high mannose species were found in Remicade^®^ (Figure 6).

More importantly, the observed differences in expressed glycan species were found to be consistent among the three analytical methods, and each technique showed good correlations in the relative abundance levels. However, for the determination of the high mannose glycan species (here, only the M5 glycoform) using the RFMS method, it should be taken into account that co-elution of the M5 and G1F-N species can cause an overestimation in the relative abundance level. As mentioned previously, the obtained values can be corrected by using the MS intensities of both species to dissect the FLD peak area and provide more accurate relative abundance levels. In addition, it is worth mentioning that the level of sialylation could not be determined using the reference 2-AB approach. In fact, potential degradation of sialylated glycans has already been observed and generally occurs during this labelling procedure. In addition, some SPE protocols are more effective than others at recovering sialylated species. Both of these issues were likely to have impacted the quality of data in this analysis, yet they are commonly encountered by practitioners and therefore believed to be reflective of a standard 2-AB analysis [11,39]. Fortunately, analysis using the RFMS label (and its associated derivatization and SPE techniques) and the analysis on subunit level samples enabled the relative quantification of the sialylated species in the infliximab products.

Altogether, the results obtained for each method were consistent with previously published data on the glycan differences between the infliximab innovator and its related biosimilar products [33,34,35]. Furthermore, these data reinforced the potential of the middle-up approach for relative glycan quantitation in biosimilar development or lot-to-lot comparisons.

## 4. Conclusions

The performance of middle-up HILIC-MS analysis for the identification and quantification of the glycosylation pattern of therapeutic mAbs was critically evaluated in comparison to two frequently practiced reference methods (released *N-*glycan analysis with 2-AB and RFMS labeling). The workflow was first illustrated through the relative quantification of the glycosylation pattern of adalimumab. An excellent correlation was found between the glycan abundance levels obtained from integration of glycoform subunit XICs (with RSD values under 0.5% for all detected scFc glycoforms) and the glycan abundance levels obtained from integration of FLD profiles related to released and 2-AB labelled glycan moieties. In addition, a good fit was observed between the values for several important *N-*glycan related CQAs, such as high mannose containing species, galactosylated species and afucosylated species in adalimumab. With this for motivation, the workflow was applied to multiple glyco-engineered mAb products, namely atezolizumab (aglycosylated), benralizumab (low fucose) and obinutuzumab (bisecting and low fucose), as well as two biosimilars (Remsima^®^ and Inflectra^®^), in comparison to their originator product infliximab (Remicade^®^). Differences in expressed glycan species were found to be consistent among the three analytical methods, and each technique showed good correlations in the relative abundance levels, even though the middle-up HILIC-HRMS glycan profiling was found to be more suitable for performing the relative quantification of co-eluting species or to prevent the degradation of sialylated glycans. Overall, these results show that HILIC-MS analysis performed at the middle-up level not only provides a means for a fast, qualitative evaluation of mAb glycosylation profiles, but also provides the basis for a robust relative quantification technique.

## Figures and Tables

**Table 1:** Comparison of relative *N-*glycan abundance levels of adalimumab. Relative abundance levels were calculated for 2-AB and RFMS labelled *N-*glycans by integration of FLD and MS detection profiles. For middle-up level analysis, integration of FLD detection profiles and integration of extracted ion chromatograms (XIC) was used for quantitation. Relative abundance levels were displayed in percentage with the standard deviations in parentheses (*n* = 3). G1F corresponds to galactosylation on the 6-branch and G1F’ to the galactose attached on the 3-branch of the glycan structure. n.d., not detected. n.q., not quantified. For the MS (XIC) column, quantitation of the G1F isomers separately was not possible due to their identical masses.

Glycan Species	2-AB		RFMS		Middle-Up	
	FLR	MS	FLR	MS	FLR	MS (XIC)
G0F	71.5 (0.4)	n.q.	70.3 (0.3)	69.8 (1.3)	64.7 (2.3)	71.5 (0.4)
G1F	10.3 (0.1)	n.q.	10.3 (0.1)	9.9 (0.4)	12.7 (0.2)	15.2 (0.4)
G1F’	4.3 (<0.1)	n.q.	4.3 (0.1)	4.3 (0.3)	3.5 (0.1)	n.q.
M5	4.6 (0.4)	n.q.	6.1 (<0.1)	6.8 (<0.1)	8.3 (0.3)	4.7 (0.3)
G0F-N	3.3 (0.1)	n.q.	3.8 (<0.1)	4.2 (0.2)	4.6 (0.6)	2.8 (<0.1)
M6	2.3 (0.2)	n.d.	2.3 (<0.1)	2.3 (0.1)	3.3 (0.4)	2.2 (0.2)
G1F-N	1.3 (<0.1)	n.d.	0.2 (<0.1)	n.d.	n.d.	1 (0.2)
G2F	1.0 (<0.1)	n.d.	1.0 (<0.1)	1.0 (0.1)	1.5 (0.4)	0.7 (0.1)
G0	0.9 (<0.1)	n.d.	0.9 (<0.1)	0.9 (<0.1)	n.d.	0.9 (<0.1)
M7	0.5 (<0.1)	n.d.	0.9 (<0.1)	1.0 (0.3)	1.5 (0.4)	1.0 (0.1)

**Table 2:** Relative glycan quantification at the subunit level using the extracted ion chromatogram (XIC). Calculated masses and the corresponding charge states used for the XIC of each scFc + glycoform are displayed. Retention times and relative abundance levels (in %, with standard deviation) were determined based on the integrated XIC profiles (*n* = 3). -K stands for C-terminal lysine clipping.

Name (scFc-K + Glycan)	Mass	RT	Relative Abundance		Charge States		
		(min)	(%, *n* = 3)	Stdev	12+	13+	14+
G0F	25,199.88	5.69	71.5	0.4	2101.00	1939.46	1801.00
G1F	25,362.02	6.11	15.2	0.4	2114.51	1951.93	1812.58
M5	24,971.63	5.88	4.7	0.3	2081.98	1921.90	1784.70
G0F-N	24,996.68	5.53	2.8	0.0	2084.06	1923.83	1786.48
M6	25,133.77	6.34	2.2	0.2	2095.49	1934.37	1796.28
G1F-N	25,158.83	6.01	1.0	0.2	2097.58	1936.30	1798.07
G2F	25,524.16	6.59	0.7	0.1	2128.02	1964.40	1824.16
G0	25,053.74	5.54	0.9	0.1	2088.82	1928.22	1790.56
M7	25,295.91	6.78	1.0	0.1	2109.00	1946.85	1807.86

## Data Availability

Data are contained within the article or supplementary material.

## Supplementary Materials

The following are available online at https://www.mdpi.com/article/10.3390/pharmaceutics13111744/s1, Figure S1: Correction of the FLD relative abundance level of co-eluting gly-coforms after RFMS labelling and HILIC-FLD-MS analysis, Figure S2: Charge state distribution of the scFc + G0F subunit in HILIC-MS, Figure S3: 2-AB glycan profiles of infliximab products in HILIC-FLD-MS, Figure S4: RFMS glycan profiles of infliximab products in HILIC-FLD-MS, Table S1: HILIC-MS analysis of *IdeS* digested and DTT reduced adalimumab, Table S2: Relative glycan quantification of benralizumab on subunit level using the XIC; Table S3: Relative glycan quantification of obinituzumab on subunit level using the XIC.
