# Supplementary material for: Quantitative N-Glycan Profiling of Therapeutic Monoclonal Antibodies Performed by Middle-Up Level HILIC-HRMS Analysis
Source: Pharmaceutics. 2021 Oct 20;13(11):1744. doi: 10.3390/pharmaceutics13111744 (PMC8617915; doi:10.3390/pharmaceutics13111744)
Supplement: Supplementary file 1 [file pharmaceutics-13-01744-s001.zip › pharmaceutics-1391064-supplementary.pdf]

pharmaceutics

Supplementary Materials: Quantitative Middle-Up Level Analysis Using HILIC-HRMS for *N*-Glycan Profiling of Therapeutic Monoclonal Antibodies

Bastiaan L. Duivelshof, Steffy Denorme, Koen Sandra, Matthew A. Lauber, Xiaoxiao Liu, Alain Beck and Davy Guillarme, Valentina D'Atri

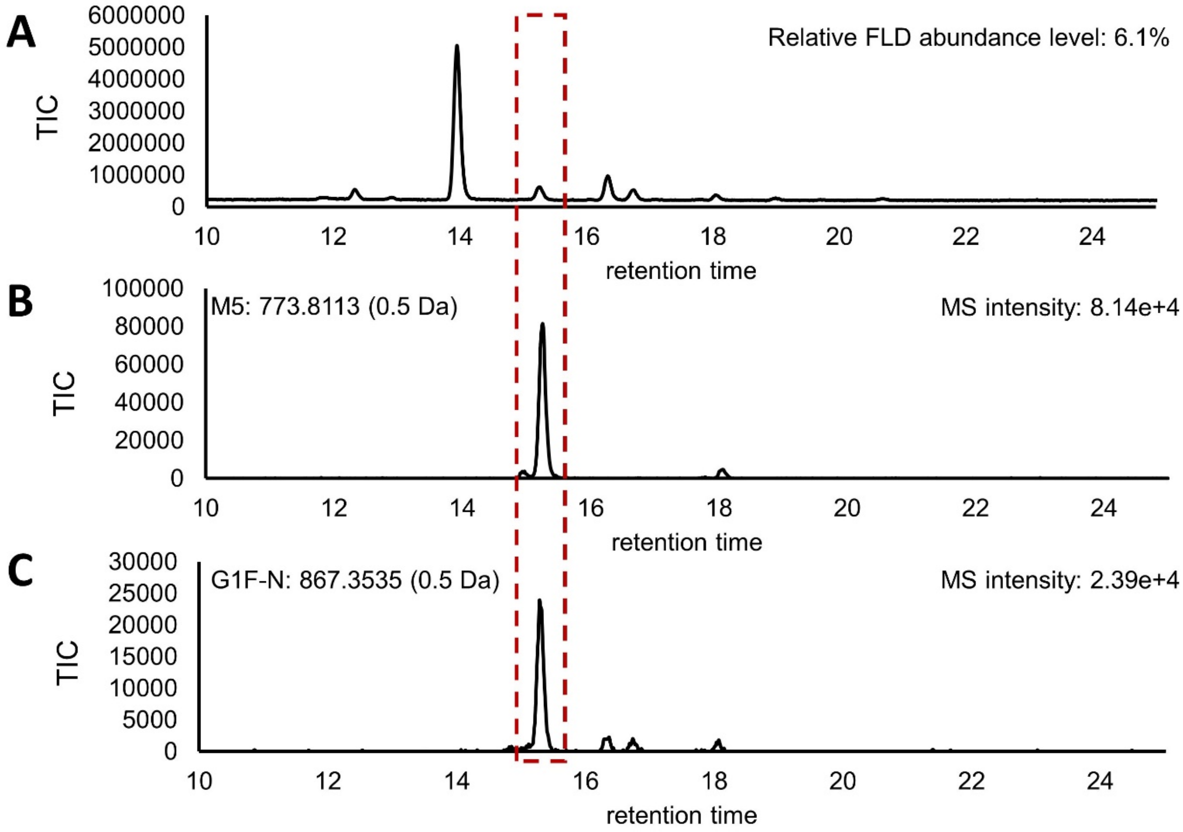

Figure S1. Correction of the FLD relative abundance level of co-eluting glycoforms after RFMS labelling and HILIC-FLD-MS analysis. (**A**) TIC profile of the RFMS labelled glycans of adalimumab with the relative FLD abundance level of the peak corresponding to the co-eluted M5 and G1F-N glycoforms (red box). (**B**) Extracted ion chromatogram (XIC) of the M5 glycan and corresponding MS intensity. (**C**) XIC of the G1F-N glycan and corresponding MS intensity.

Table S1. HILIC-MS analysis of *IdeS* digested and DTT reduced adalimumab. Peak retention times and mass assignment. –Kclip stands for C-terminal lysine clipping (-128 Da). + Gly stand for glycation (+ 162 Da).

rt (min)	Trivial assignment	Theoretical masses (Da)	Experimental masses (Da)	Δm (Da)	ppm
3.34	Fd	25454.25	25453.32	0.93	36.55
4.76	LC	23407.82	23406.96	0.86	36.77
5.16	LC + Gly	23569.82	23569.23	0.59	25.06
5.53	scFc-Kclip G0	25053.74	25052.54	1.20	47.73
5.53	scFc-Kclip G0F-N	24996.68	24995.83	0.85	34.18
5.68	scFc -Kclip G0F	25199.88	25199.09	0.79	31.23
5.88	scFc -Kclip M5	24971.63	24970.72	0.91	36.52
6.10	scFc -Kclip G1F	25362.02	25360.97	1.05	41.30
6.34	scFc-Kclip M6	25133.77	25132.72	1.05	41.88
6.59	scFc -Kclip G2F	25524.16	25523.73	0.43	16.77
6.79	scFc-Kclip M7	25295.91	25294.01	1.90	75.23

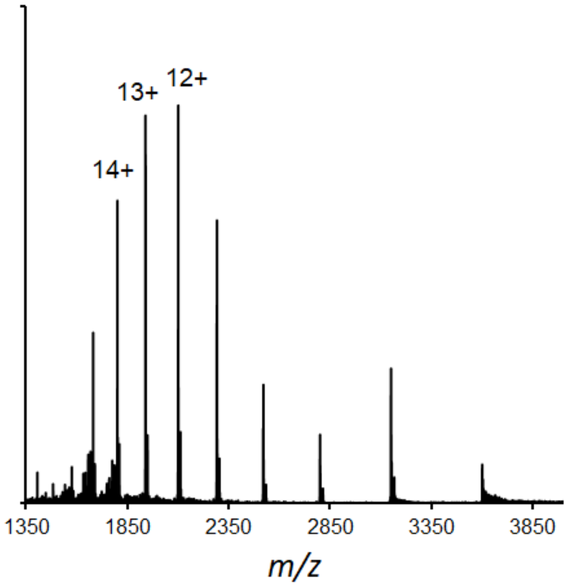

Figure S2. Charge state distribution of the scFc + G0F subunit in HILIC-MS. Mass spectra of the peak corresponding to the scFc +G0F subunit of adalimumab.

Table S2. Relative glycan quantification of benralizumab on subunit level using the extracted ion chromatogram (XIC). Retention times and relative abundance levels (in %, with standard deviation) are determined based on the integrated XIC profiles (n = 3). –Kclip stands for C-terminal lysine clipping.

Component name	Observed RT (min)	Response	Relative abundance	STDEV
scFc-Kclip G0	5.40	945156.00	84.28	0.33
scFc-Kclip G1	5.82	100994.00	9.01	0.14
scFc-Kclip M5	5.80	30013.33	2.67	0.19
scFc-Kclip G0-N	5.24	24232.33	2.16	0.05
scFc-Kclip G1-N	5.40	18981.33	1.69	0.10
scFc-Kclip G2	6.29	2172.00	0.19	0.01

Table S3. Relative glycan quantification of obinituzumab on subunit level using the extracted ion chromatogram (XIC). Retention times and relative abundance levels (in %, with standard deviation) are determined based on the integrated XIC profiles (n = 3).

Component name	Observed RT (min)	Response	Relative abundance	STDEV
scFc G0FB	5.86	260917.67	25.01	0.59
scFc G0B	5.68	228549.67	21.93	0.15
scFc G1FB	6.20	120582.33	11.56	0.43
scFc G2	6.24	102676.67	9.85	0.48
scFc G1	5.83	68350.67	6.54	0.40
scFc G1B	6.02	67878.67	6.53	0.57
scFc G0	5.49	43485.00	4.17	0.07
scFc G0F	5.68	38302.33	3.67	0.14
scFc M5	5.81	36283.00	3.48	0.07
scFc G1F	6.07	34325.67	3.30	0.14
scFc G2F	6.44	23639.33	2.27	0.21
scFc G2B	6.20	17477.00	1.67	0.31
		**Bisecting glycans**	66.71	
		**Afucosylation**	50.71	

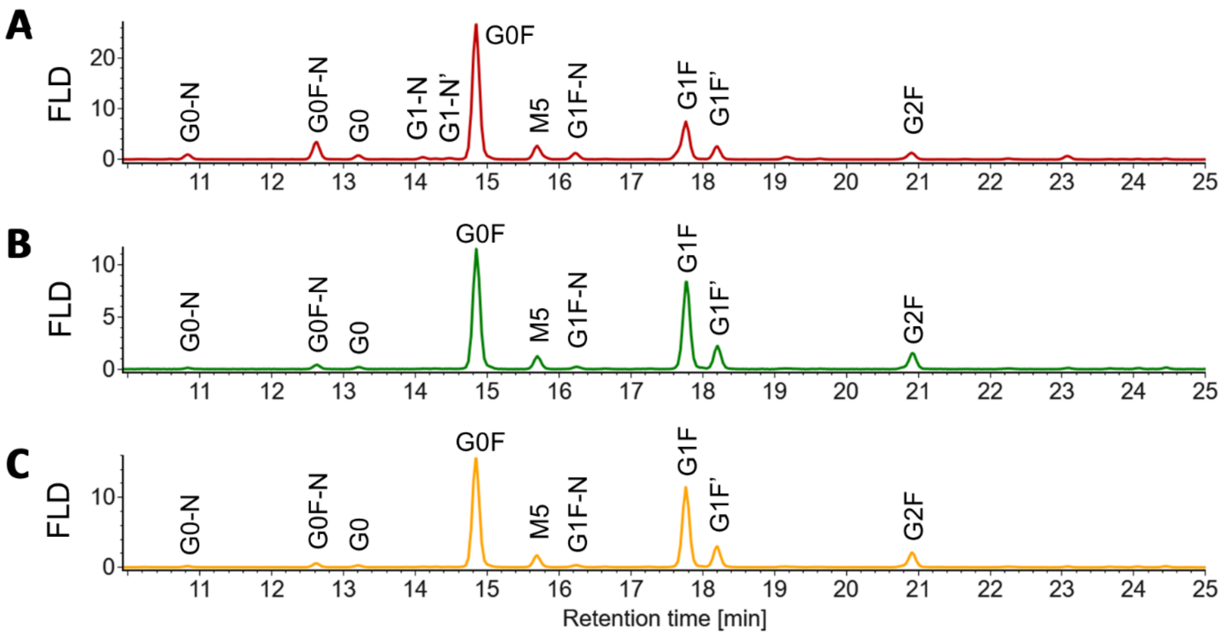

Figure S3. 2-AB labelled glycan profiles of infliximab products in HILIC-FLD-MS. Fluorescence chromatograms of the N-glycan profile of Remicade® (**A**), Remsima® (**B**) and Inflectra® (**C**).

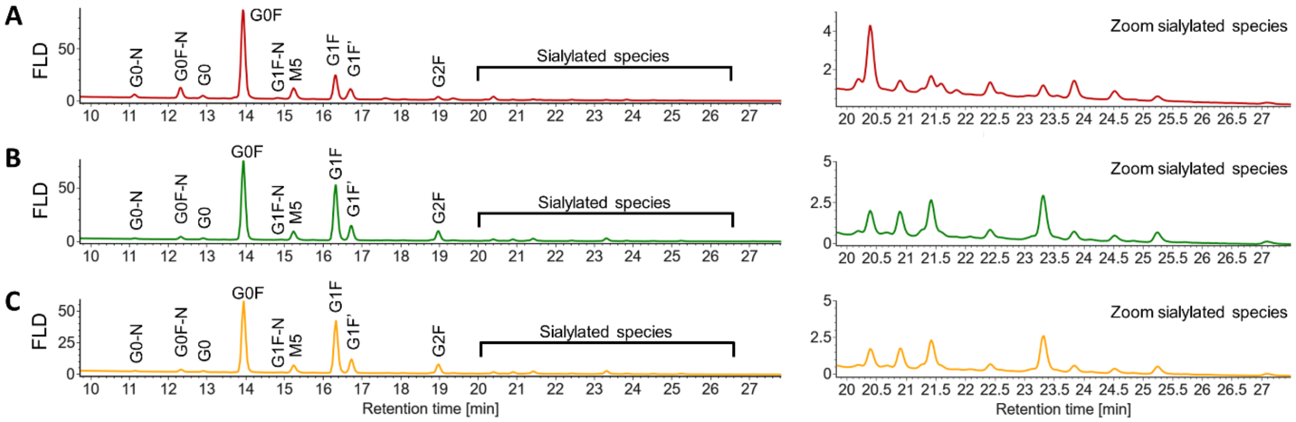

Figure S4. RFMS labelled glycan profiles of infliximab products in HILIC-FLD-MS. Fluorescence chromatograms of the N-glycan profile of Remicade® (**A**), Remsima® (**B**) and Inflectra® (**C**).
